# BisQC: an operational pipeline for multiplexed bisulfite sequencing

**DOI:** 10.1186/1471-2164-15-290

**Published:** 2014-04-16

**Authors:** Gary G Chen, Alpha B Diallo, Raphaël Poujol, Corina Nagy, Alfredo Staffa, Kathryn Vaillancourt, Pierre-Eric Lutz, Vanessa K Ota, Deborah C Mash, Gustavo Turecki, Carl Ernst

**Affiliations:** 1 Department of Psychiatry, McGill University, Douglas Hospital Research Institute, Montreal, Quebec, Canada; 2 McGill Group for Suicide Studies, Douglas Hospital Research Institute, 6875 LaSalle Boulevard, Frank Common Building, Room 2101.2 Verdun, Montreal, QC, H4H 1R3, Canada; 3 McGill University and Genome Quebec Innovation Center, Montreal, Quebec, Canada; 4 Departments of Neurology and Molecular and Cellular Pharmacology, Miller School of Medicine, Miami, Florida, USA; 5 Department of Human Genetics, McGill University, Montreal, Quebec, Canada

**Keywords:** DNA methylation, Bisulfite sequencing, Bioinformatics

## Abstract

**Background:**

Bisulfite sequencing is the most efficient single nucleotide resolution method for analysis of methylation status at whole genome scale, but improved quality control metrics are needed to better standardize experiments.

**Results:**

We describe BisQC, a step-by-step method for multiplexed bisulfite-converted DNA library construction, pooling, spike-in content, and bioinformatics. We demonstrate technical improvements for library preparation and bioinformatic analyses that can be done in standard laboratories. We find that decoupling amplification of bisulfite converted (bis) DNA from the indexing reaction is an advantage, specifically in reducing total PCR cycle number and pre-selecting high quality bis-libraries. We also introduce a progressive PCR method for optimal library amplification and size-selection. At the sequencing stage, we thoroughly test the benefits of pooling non-bis DNA library with bis-libraries and find that BisSeq libraries can be pooled with a high proportion of non-bis DNA libraries with minimal impact on BisSeq output. For informatics analysis, we propose a series of optimization steps including the utilization of the mitochondrial genome as a QC standard, and we assess the validity of using duplicate reads for coverage statistics.

**Conclusion:**

We demonstrate several quality control checkpoints at the library preparation, pre-sequencing, post-sequencing, and post-alignment stages, which should prove useful in determining sample and processing quality. We also determine that including a significant portion of non-bisulfite converted DNA with bisulfite converted DNA has a minimal impact on usable bisulfite read output.

## Background

DNA methylation has a role in the development of eukaryotic organisms [1,2] and may represent the interface between genome and environment [3]. Currently, the most widely used method for detecting 5-methylcytosine is the treatment of DNA with sodium bisulfite [4], which results in the deamination of all non-methylated cytosine to uracil. Since sodium bisulfite does not convert 5-methylcytosine bases to uracil, this approach allows for the direct interpretation of where methylation has or has not occurred in the genome. This interpretation is complicated by the need to generate percentage methylation statistics per cytosine residue because the same locus can have different methylation levels across cells. This complication underscores the need to have careful metrics to assess experimental procedures.

Bisulfite treatment combined with Next-Generation sequencing (NGS) is the method of choice for the NIH Epigenomics Roadmap [5], a project with a stated goal to map methylation patterns in multiple tissue types. This experimental design underlies the expectation that there are different methylation patterns in different tissues, and there is a high likelihood that cells that make up these tissues themselves have different methylation patterns, even at the same genomic loci. This variation complicates analysis and can lead to high levels of noise, making data interpretation challenging. One major issue for all bis-DNA Next Generation sequencing (BisSeq) experiments is read alignment to a reference genome. This is due to the loss of unmethylated C residues (observed as T residues after sequencing) leading to decreased complexity. Specifically, the longer and more diverse a sequenced read, the more likely it is to align to the genome. Loss of base diversity from the decrease in C bases means that individual reads may appear to align to multiple regions of the genome. A second major reason for alignment difficulties is the diversity of methylation patterns at cytosine loci. In a read containing many C residues, methylation patterns at the same base could be different between reads. This means that reads from the same genomic locus could align to different genomic regions.

Bisulfite conversion of DNA followed by massively parallel sequencing is likely to be the most practical approach to map methylation in the coming years, whether in reduced or complete genomic space [6,7]. Reduced-Representation bisulfite sequencing (RRBS) uses an MspI digestion (cutting at C^V^CGG) prior to bisulfite conversion and library preparation to reduce genomic space, resulting in the sequencing of ~2.5% of the genome; however, a thorough analysis of its limitations, and methods to cope with these inadequacies, has not been fully addressed. Early protocols for bisulfite sequencing [8-10] were described for the Illumina Genome Analyzer IIx sequencer, where library preparation was singleplexed. Later protocols [11] switched to multiplexed indexing approaches using Illumina TrueSeq DNA sample preparation. Notably, early protocols focused on library construction, while recent protocols have explored strategies for successful sequencing and analysis [12]; however, there currently lacks a simple, detailed description of multiplex sequencing using conventional tools; complex, hard-to access strategies are exemplified by the proposal to use ‘Dark’ sequencing [11] in bisulfite sequencing experiments.

The purpose of the current work is to provide a simple BisSeq protocol, with several QC checks throughout that can be used in standard laboratories and to test two main questions: 1) What is the function of pooling non-bisulfite DNA with bisulfite converted DNA in a single lane, and 2) What is the validity of using duplicate reads to calculate coverage statistics? In this work we carefully and completely lay out experimental procedures and QC parameters to assess BisSeq experiments and we suggest that pooling 30% non-bisulfite converted DNA with bisulfite converted DNA has a minimal effect on bisulfite read output, meaning that sequencing non-bisulfite DNA samples from unrelated experiments is practical. We find also that using duplicate reads to calculate coverage is legitimate, but that a simple test to assess whether the total read pool is representative of a read pool with no duplicates should be applied first.

## Methods

### Library preparation

**Figure 1:**
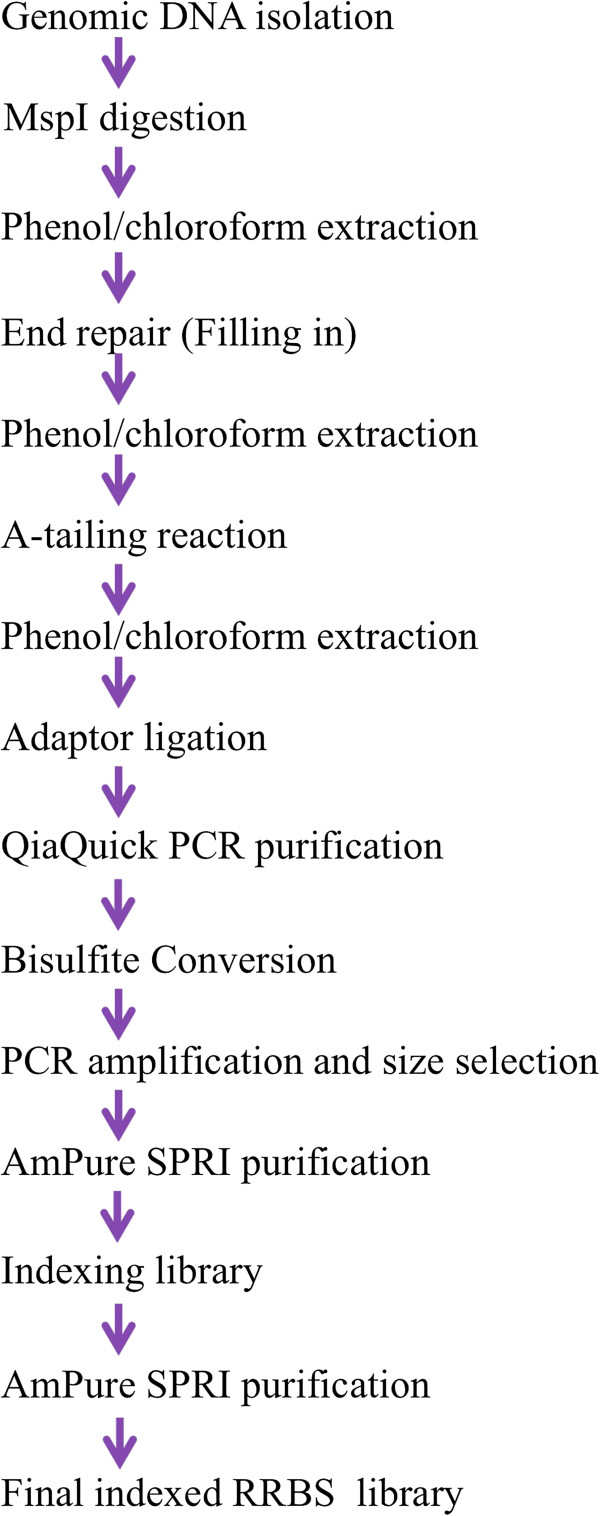
Flow chart of library preparation steps.

**Figure 2:**
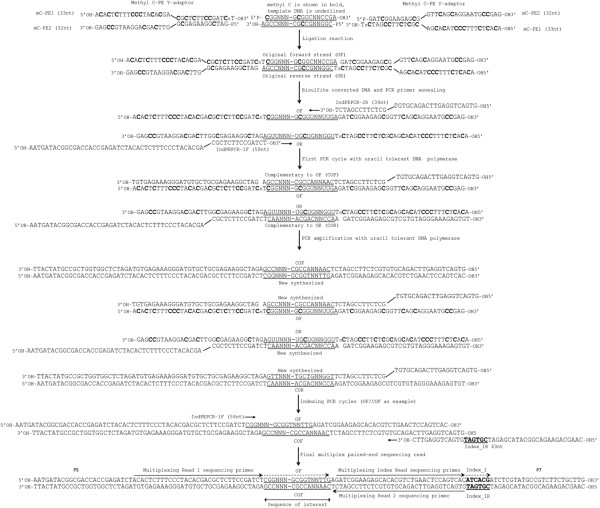
**Major RRBS library construction steps.** This figure demonstrates adaptor ligation (step 1) and barcode indexing (step 2) for Illumina two-step library preparation, as well as in between steps including bisulfite treatment. We show how the 2-step procedure affects DNA inserts when used for RRBS directional sequencing. First, DNA inserts (underlined) with ‘A’ overhangs are ligated to methylated Illumina adaptors (methylated cytosines are marked in bold), meC-PE1 and meC-PE2. Next, adaptor ligated-DNA inserts are bisulfite treated and amplified using primer indPEPCR1F and indPEPCR2R. All unmethylated cytosines deaminate to uracil. We show two cycles of the PCR reaction toamplify bisulfite fragments to show how DNA inserts change after bisulfite treatment and amplification, as well as to track original top (OT) and original bottom (OB) strands. After an appropriate number of cycles (appropriate is defined by the visualization of bands shown in this manuscript in the library preparation stage), bisulfite treated libraries can be indexed, then sent for sequencing. Note that for directional sequencing all sequencing reads are either from the original top (OT) or the original bottom (OB) strands. The first three bases of almost all RRBS reads are either CGG or TGG, depending on their genomic methylation state and this applies to reads generated from both OT and OB strand. Therefore almost every read in a directional RRBS sequencing experiment that use MspI digestion contains at least one CpG at the 2nd and 3rd base positions, plus any internal CpGs (provided they are not in CCGG or CCGG sequences). Internal CpGs can be in CCGG sequence where MspI does not cut when the first C is methylated. Abbreviations: C (Bold): methylated C; p: phosphate; s: phosphorothioate bond. Illustrated insert DNA is underlined. P5 (5′ AATGATACGGCGACCACCGA 3′) and P7 (5′ CAAGCAGAAGACGGCATACGA 3′) are flow cell attachment sites.

All tissue samples used in this study were provided by the Brain Endowment Bank™ following protocols approved by the research ethics board of the University of Miami Miller School of Medicine. Brain samples (anterior caudate nucleus) were obtained at autopsy following the principles of the Helsinki declaration and next-of-kin gave written informed consent. We used NEBNext Illumina Library Prep Master Mix kit for the library construction work. All experiments herein use reduced representation bisulfite sequencing; however, most optimizations can be applied to standard bisulfite sequencing. Figure 1 shows a flow chart of library preparation procedures for multiplexed Reduced-Representation Bisulfite Sequencing (mRRBS), which takes on average 7–10 days. Figure 2 shows a molecular level illustration of library preparation after the adaptor ligation stage.

### Enzyme digestion

**Figure 3:**
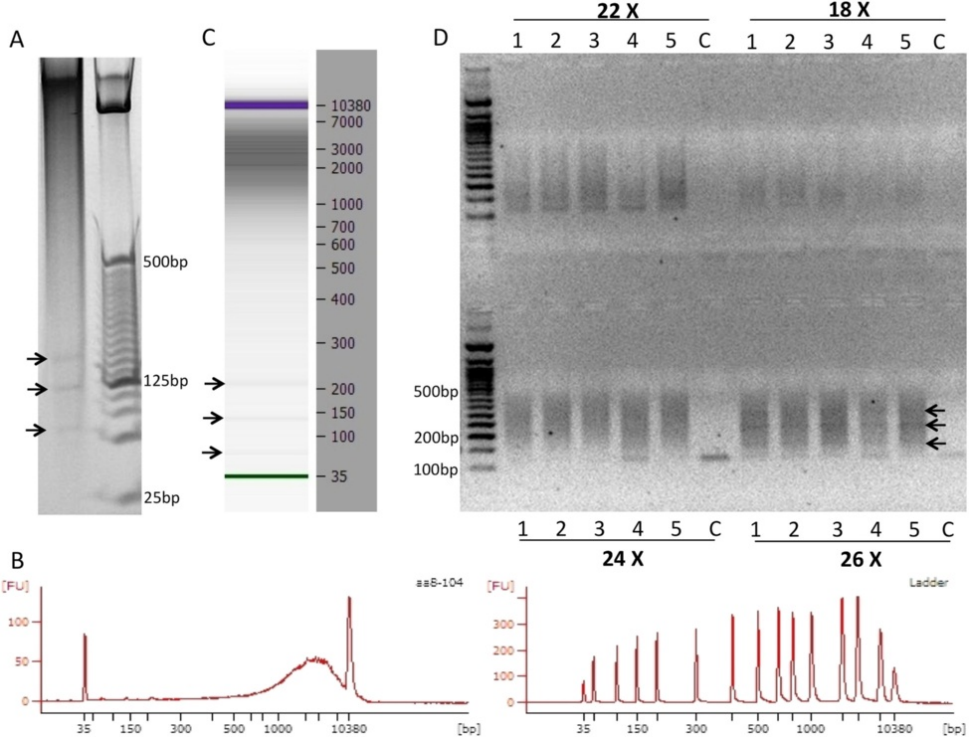
**Standards for MspI digestion and progressive PCR. A)** MspI digestion of human genomic DNA isolated from human post-mortem brain tissues. DNA (200 ng) was digested by MspI and run on a 4–20% precast polyacrylamide gel and stained with EtBr. Arrows show three satellite DNA bands characteristic of this enzymatic digestion. **B)** Agilent 2100 Bioanalyzer chromatogram of MspI digested genomic DNA. **C)** Bioanalyzer 2100 image of a single library from an MspI digested DNA sample. Notice that the satellite bands (indicated by arrows) are still visible on the Bioanalyzer image. **D)** Progressive PCR amplification combined with limited PCR extension time allows for size selection and amplification of six bisulfite converted libraries (Lanes 1–5 are distinct RRBS libraries; lane 6 (‘C’) is a negative control). After different progressive PCR cycles (18X, 22X, 24X, or 26X – the same libraries are shown for each cycle number) band intensity increases as cycle number increases. Arrows indicate the three satellite DNA bands that are still visible in these libraries.

After DNA purification using the QIAamp genomic DNA micro isolation kit from human brain, 2-5 μg of genomic DNA was used to carry out the MspI (New England Biolabs) digestion at 37°C for 7 hours using 20 units of enzyme per μg of DNA. Digested DNA was purified by phenol/chloroform (p/c) extraction (49:49:2; phenol:chloroform:isoamyl alcohol). The aqueous top layer containing genomic DNA was precipitated in the presence of NaCl (0.3 M final) and glycogen (25 μg final). The precipitated DNA was pelleted via centrifugation and washed with 500 μl 80% ethanol (rinsing the pellet instead of re-suspending) and then centrifuged again at 12000 rpm for 20 minutes to solidify the pellet. The pellet containing MspI fragmented DNA was resuspended in 100 μl dH_2_0 for the end-repair reaction. Five-ten percent of the purified MspI fragmented DNA was then run on a precast 4-20% gradient polyacrylamide TBEx1 gel and stained with ethidium bromide (EtBr; Invitrogen). This is an important step to verify the enzymatic digestion of DNA; a complete MspI digestion will produce visible satellite bands in a smearing background (Figure 3). A high sensitivity DNA chip to check the completion of MspI digestion can also be used (Figure 3C).

### End repair (Filling-in and dA-tailing)

MspI recognizes double stranded DNA at 5′-C^CGG-3′ and cleaves the phosphodiester bonds upstream of CpG dinucleotide. This reaction results in DNA fragments with 5′ overhangs, so end repair is necessary to fill-in the 3′ termini of each fragment. This way, all MspI digested library fragments should contain a CpG dinucleotide on both ends of the fragment. The NEBNext DNA Library Prep Master Mix Set for Illumina separates the filling-in step from dA-tailing reaction.

### Filling- in of MspI rragmented DNA

Using the MspI fragmented DNA (40–85 μL) described above, the NEBNext End Repair Reaction Buffer (10X; 10 μL), and the NEBNext End Repair Enzyme Mix (5 μL), we incubated the solution (final volume of 100 μl) at 20°C for 30 minutes. After incubation, the reaction mixture was diluted to 200 μL with dH_2_O. Next, we added 200 μL of p/c at room temperature. After ethanol precipitation, DNA was resuspended in 50 μL dH_2_O in preparation for the dA-tailing reaction.

### dA-tailing of blunt end MspI fragment

The enzymatic process that adds an extra adenosine (A) to both the plus and minus strand 3′ termini is referred to as dA-Tailing and is necessary for ligation of the adaptors (which contain a 3′ dT overhang). Following the NEBNext DNA library Prep Master Mix Set for Illumina kit manual, we carried out the dA-Tailing reaction. We used 42 μL blunt-end DNA, 5 μL of NEBNext dA-Tailing Reaction Buffer (10X), and 3 μL Klenow Fragment (3′- > 5′ exo-), for a total volume of 50 μL and incubated at 37°C for 30 minutes. Next, p/c extraction and ethanol precipitation were performed following the same steps as outlined above, without the inclusion of glycogen (no glycogen is needed, since previously added glycogen is co- precipitated with genomic DNA). The final volume of dA-tailed MspI fragment is 30 μl in dH_2_O.

### Methylated adaptor design

We used the two-step Illumina adaptor design for the adapters and the PCR indexing primers because it decouples the indexing reaction from library amplification (Figure 2), and allows for more efficient bisulfite treated DNA library amplification and size selection. We synthesized published Illumina paired-end adaptor oligonucleotides, and had all cytosines replaced with 5′methyl-cytosines in order to prevent the deamination of the adaptor cytosines in the bisulfite conversion reaction. All adaptors and indexing primers are listed in Table 1.

**Table 1:** Sequences and specific modifications of oligonucleotides used in the BisQC protocol

**Name**	**Sequence (5′ to 3′)**
Methylated C adaptor: mC-PE1	A**C**A**C**T**C**TTT**CCC**TA**C**A**C**GA**C**G**C**T**C**TT**CC**GAT**C**sT-OH
Methylated C adaptor: mC-PE2	p-GAT**C**GGAAGAG**C**GGTT**C**AG**C**AGGAATG**CC**GAG-OH
PCR primer: IndPEPCR_F	AATGATACGGCGACCACCGAGATCTACACTCTTTCCCTACACGACGCTCTTCCGATCsT
PCR primer: IndPEPCR_R	GTGACTGGAGTTCAGACGTGTGCTCTTCCGATCsT
PE-qPCR_F (Phix-PE-qPCR_F)	AATGATACGGCGACCACCGA-OH
PE-qPCR_R (Phix-PE-qPCR_R)	CAAGCAGAAGACGGCATACGA-OH
Index_1R primer	CAAGCAGAAGACGGCATACGAGAT*CGTGAT*GTGACTGGAGTTC-OH
Index_2R primer	CAAGCAGAAGACGGCATACGAGAT*ACATCG*GTGACTGGAGTTC-OH
Index_3R primer	CAAGCAGAAGACGGCATACGAGAT*GCCTAA*GTGACTGGAGTTC-OH
Index_4R primer	CAAGCAGAAGACGGCATACGAGAT*TGGTCA*GTGACTGGAGTTC-OH
Index_5R primer	CAAGCAGAAGACGGCATACGAGAT*CACTGT*GTGACTGGAGTTC-OH
Index_6R primer	CAAGCAGAAGACGGCATACGAGAT*ATTGGC*GTGACTGGAGTTC-OH
Index_7R primer	CAAGCAGAAGACGGCATACGAGAT*GATCTG*GTGACTGGAGTTC-OH
Index_8R primer	CAAGCAGAAGACGGCATACGAGAT*TCAAGT*GTGACTGGAGTTC-OH
Index_9R primer	CAAGCAGAAGACGGCATACGAGAT*CTGATC*GTGACTGGAGTTC-OH
Index_10Rprimer	CAAGCAGAAGACGGCATACGAGAT*AAGCTA*GTGACTGGAGTTC-OH
Index_11Rprimer	CAAGCAGAAGACGGCATACGAGAT*GTAGCC*GTGACTGGAGTTC-OH
Index_12Rprimer	CAAGCAGAAGACGGCATACGAGAT*GAACAT*GTGACTGGAGTTC-OH

*Abbreviations*: ***C**** (Bold)* methylated C, *p* phosphate, *s* phosphorothioate bond.

### Illumina methylated Y-adaptor annealing

Prior to adaptor ligation, we carried out an adaptor Y fork annealing reaction by combining equal molar ratios of methylated PE1 and methylated PE2 adaptors. With this, annealed adaptor oligonucleotides can be kept at -20°C for many months before use, provided high temperature and other denaturing conditions are avoided. To perform this reaction, we mixed 50 μL each of mC-PE1 (25 μM) and mC-PE2 (25 μM) in PCR well-plates and carried out the following denaturing and annealing reaction on the thermal cycler: 95°C, 120 s; 80°C, 60 s; 70°C, 60 s; 60°C, 60 s; 50°C, 60 s; 40°C, 60 s; 30°C, 60 s; 4°C indefinitely.

### Ligation of methylated Y-adaptor to dA-tailed DNA fragments

For the ligation reaction, we used 25 μL dA-tailed DNA, 10 μL NEB Quick ligation Reaction Buffer (5X), 10 μL pre-annealed Illumina methylated Y-adaptors, and 5 μL NEB Quick T4 ligase for a total volume of 50 μL. This was then incubated for 1 hour at 16°C, followed by a second incubation for 30 minutes at 20°C.

### Purification of adaptor- ligated library

We used the QiaQuick PCR purification kit for the purification of the adaptor-ligated libraries. We used 250 μL of QIA buffer PE to carry out the purification process for 50 μL of the ligation mixture. The purified libraries were then eluted in 100 μL of dH_2_O.

### Bisulfite conversion

We used the Qiagen EpiTect Fast 96 Bisulfite Kit to carry out the bisulfite conversion of adaptor-ligated library. This single conversion step is reported to be sufficient to achieve a ≥99% conversion rate (discussed in more detail in the results section). We used 50% of the purified adaptor ligated library for bisulfite conversion. To purify DNA, the reaction mixture was transferred into a 96-well plate, with a high affinity membrane on the bottom of each well (Qiagen). Buffer BL, bisulfite conversion mixture and ethanol (96%) were added sequentially, mixed, then left to stand for 2 minutes. After spinning, single stranded, bis-converted DNA was bound to the membrane. We then washed twice with buffer BW, than twice with buffer BD to achieve a complete on-membrane desulfonation. This was followed by two more washes with BW then a final elution with buffer EB.

### PCR amplification of bisulfite converted libraries

Agilent Pfu turbo Cx Hotstart DNA polymerase has the property of uracil-tolerance and high fidelity DNA polymerization. Amplification of bisulfite-converted libraries was carried out simultaneously with the library size selection process by using the progressive PCR method. IndPEPCR_F (33 nt) and _R (32 nt) (Table 1 and Figure 2) were used as PCR primers.

The detailed PCR reaction mixture (200 μL volume) is as follows:

10X pfu Turbo Cx Rxn Buffer (Agilent)-20 μL

dNTP (10 mM each)-4 μL

IndPEPCR_F (25 M)-1 μL

IndPEPCR_R (25 M)-1 μL

Pfu Turbo Cx Hot Start DNA Polymerase-2 μL

Bisulfite converted RRBS library-50 μL

dH_2_O -122 μL

The Pfu Turbo Cx Hot Start DNA Polymerase should be added last. The detailed amplification cycle is as following:

1. 95°C-90 s

2. 95°C-30 s

3. 60°C-30 s

4. 72°C-30 s

5. 4°C indefinitely (in 4°C fridge, do not freeze the PCR reaction)

Repeat steps 2–4 for 18 cycles

**Figure 4:**
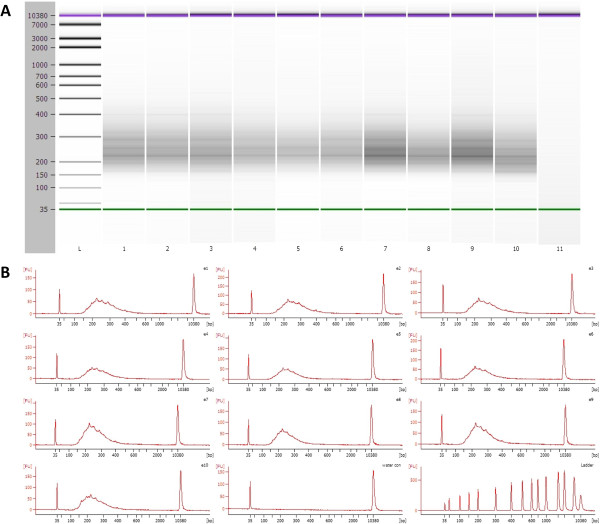
**Agilent 2100 Bioanalyzer images from final reduced representation bisulfite libraries. A)** High Sensitivity DNA Chip from 10 RRBS libraries. Notice that the satellite bands are still visible on the Bioanalyzer gel image **B)** Chromatogram representation of panel A showing high quality RRBS libraries.

The final PCR products represent the minimally-amplified and size-selected non-indexed RRBS libraries. We confirmed the correct size amplification by running a 2.0 % HR Agarose Gel (100 mL 1XTAE + 2.0 g of HR Agarose + 2.5 μL of 10mg/mL EtBr) or an Invitrogen 4-20% gradient polyacrylamide gel (1XTAE), or Invitrogen E-gel 2% with SYBR Safe, all after 18 cycles of PCR. Samples showing faint but visible 150–400 base pair (bp) smearing on the gel have the optimal amplification PCR cycles. Satellite DNA bands should also be visible in the smearing background for a well-constructed and optimally amplified RRBS library (Figure 3). Samples that give very faint smearing require additional PCR amplification cycles. Samples requiring extra PCR cycles (using the PCR reaction tubes kept at 4°C) can be returned to the thermal cycler for 2–4 more cycles of amplification following the above progressive PCR protocol. Ten μL of these ‘additional cycle’ amplified PCR reaction mixtures can be run on a gel and checked for smearing. Thus, final library selection is determined by visual inspection of gel images for appropriate smearing and satellite band patterns (Figure 3). This is required because of the large variation observed across libraries, even with identical starting DNA concentrations and enzyme digestion times. Finally, we perform a cleanup step with the remaining PCR products from all libraries using AMPure XP SPRI beads (Agencourt, Beckman-Coulter) using 60 μl/55 μl (beads/DNA) ratio. After purification, 1 μL of the purified library was used for quality control using an Agilent Bioanalyzer 2100 High Sensitivity DNA Chips. A good amplified size-selected and purified library produces a smear covering 150–400 bp with visible satellite bands, similar to that seen in Figure 4, with very little primer-dimers.

### Indexing

Using 50 μL of purified, non-indexed library, we performed the following PCR indexing reaction for each library. The PCR primers used for indexing reaction are IndPEPCR_F (33 nt) and Index_#R primer (43 nt)

10X pfu Turbo Cx Rxn Buffer (Agilent)-20 μL

dNTP (10 mM each)-4 μL

IndPEPCR_F (25 M)-1 μL

IndPEPCR_#R (25 M)-1 μL

Pfu Turbo Cx Hot Start DNA Polymerase-2 μL

Non-indexed RRBS library-50 μL

dH_2_O-122 μL

The Pfu Turbo Cx Hot Start DNA Polymerase should be added last.

Using the following PCR steps:

1. 95°C-90 s

2. 95°C-30 s

3. 65°C-30 s

4. 72°C-30 s

Repeat 2–4 times

5. 4°C indefinitely

We used 60μl/55μl (beads/DNA) ratio. AMPure SPRI bead-purified library was eluted in 60 μL of dH_2_O. Purified libraries can be screened on an Agilent 2100 BioAnalyzer (Figure 4A and B).

### Molecular cloning and sanger sequencing

**Figure 5:**
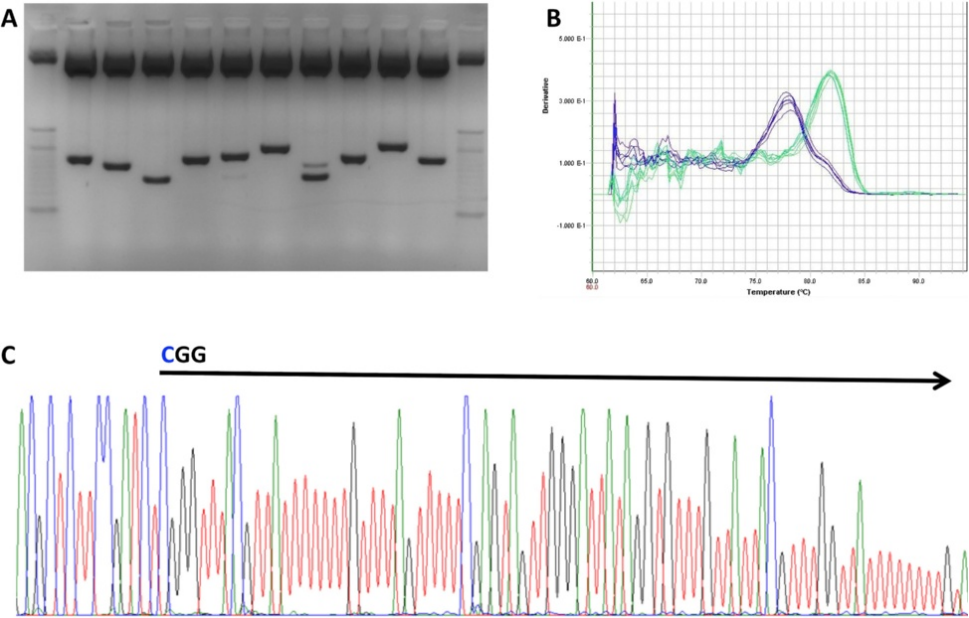
**Pre-sequencing quality control of bisulfite-converted DNA libraries. A)** Cloning and subsequent EcoRI release of library inserts reveals a random size distribution as revealed on an agarose gel. The extra band in some lanes represents enzyme cut sites present in the inserts. **B)** qPCR dissociation curve of a bisulfite- converted DNA library using primers directed at adaptors to the bisulfite library (blue peak) and PhiX standard DNA library (green peak). Bisulfite converted DNA libraries have a lower melting temperature due to loss of cytosine residues. The qPCR dissociation curve only serves as a general tool to verify bisufite conversion and is not able to distinguish a minor bisulfite conversion problem. **C)** Sanger sequencing reaction of a single clone insert from A). Plasmid is detectable by presence of cytosine residues; insert begins at CGG residue marked by the arrow. Note the lack of cytosine residues in the insert, except at CpG loci (blue ‘C’ peaks under black arrow). Base colors are: C: Blue, G: Black, T: Red, A: Green.

5 μl of AMPure bead-purified library (total 60 μl) was used for cloning experiments. The single band product was cloned using chemically competent E.coli cells and the pCR™4-TOPO® TA vector (Invitrogen Cat#450030) using 4 μl of fresh PCR product, 1 μl of vector, 1 μl of salt solution, and ligated for 5 minutes at room temperature. Four μl of ligated product was added to 50 μl of competent cells and incubated on ice for 30 minutes, 240 μl of S.O.C. medium (Invitrogen Cat#15544-034) was added to the cells incubated in a 37°C shaker for 1 hour. 50 μl of transformed cells was plated on ampicillin agar plates and incubated for 16 hours at 37°C. Prior to sequencing, we assessed some band sizes by an EcoRI digestion of plasmids (Figure 5A). Sequencing was done using rolling circular amplification, a service provided by Genewiz, Inc (South Plainfield, NJ). M13F (-21) sequenced colonies were aligned using Lasergene® SeqMan Pro (DNASTAR, Inc. Madison, WI).

For methylation assessment of a single, targeted locus, a 250 bp amplicon was isolated from the original RRBS library preparation for sample G12. Two μl of template was amplified using High Fidelity Platinum Taq (Invitrogen Cat #11304-102) and the PCRx enhancer system to facilitate amplification of CG rich template (Cat# 11495–017), with the following thermocycling conditons: 95° for 5 minutes, (95° for 30s, 58° for 30s and 72° for 45 seconds) x 45 cycles, 72° for 7 minutes, 4° indefinitely. Bisulfite primers were designed using Methyl Primer Express® (Applied Biosystems), forward 5′- GGG AAG AGT TGG TTA GAG AGA -3′ and reverse 5′-AAA ACC CCC TAT AAA AAA ACC C-3, corresponding to (HG19) Chromosome 3: 75, 718, 452–75, 718, 701.

### Massively parallel (next generation) sequencing

We used the Illumina HiSeq2000 platform, with single-end 50 cycle sequencing. Sequencing was performed by the McGill University and Génome Québec Innovation Centre in Montreal, Quebec. Data was downloaded onto our servers in FASTQ format. Illumina de-indexed data was first processed using FastQC tool v0.10.0 (http://www.bioinformatics.babraham.ac.uk/projects/fastqc/), which provides a user-friendly overview of raw sequencing data. The programs fastx_clipper, fastx_trimmer and fastx_collapser [part of the FASTX-Toolkit (http://hannonlab.cshl.edu/fastx_toolkit/)] were used to remove adaptors, filter low quality reads, and remove reads < 20 bp in length.

### Alignment

There are many excellent aligners for bisulfite sequencing data including Bismark [13], BSMAP [14,15], and RMAP [16], BS-Seekeer2 [17] and some of these have been recently assessed [18]. We opted to use Bismark [14] for its flexibility and compatibility with downstream processing tools such as MethylKit [19], which we use routinely for post-alignment processing. All user-set features were set to default, except for: --best, --n 2, and –directional. For data visualization, we wrote a custom Perl script to convert Bismark output alignment files into the SAM (Sequence Alignment/Map) format for the visualization of the alignment reads into the Integrated Genomic Viewer [20]. This script is available for download at: http://www.mcgill.ca/psychiatricgenetics/tools-0. Further description of bioinformatic details can be found in the results section.

## Results

### Post-bisulfite indexing for increased flexibility and improved library purification

Currently, most NGS bisulfite DNA protocols take advantage of the ‘one-step’ design, where indexing and adaptor ligation occur in one step, but this creates longer products (Adaptors with indexes are >100 nucleotides for the one-step design as opposed to 64 nucleotides in the two-step design) which influences clean-up procedures and allows less flexibility in library amplification – an important factor in bisulfite DNA projects because of the variability in DNA concentration after bisulfite treatment. Pre-indexing also requires decisions to be made about what samples to pool together prior to knowing the quality of each library. To devise a post-library indexing strategy, we used the Illumina two-step paired end design (Figure 2) which uses a third indexing primer (Table 1 lists the sequences for all oligonucleotides used in these experiments).

A major benefit to using the two-step Illumina adaptor/indexing technology beyond the increased flexibility, is that they form dimers less than 100 bp, allowing for the use of spin columns rather than AMPure beads; adaptor dimers using this strategy are 65 bp in contrast to the 126 bp dimers generated by the TruSeq protocol. Spin columns recover all fragments >100 bps, whereas AMPure bead purification functions best at high DNA concentrations or with small volume operations. This means that a greater proportion of the library is retained after purification. A disadvantage of using the two-step approach is that fusion products between the adaptor and indexing primer form, and this product needs to be removed (see Additional file 1 for details).

### Simultaneous amplification and size selection of bisulfite libraries by progressive PCR

Progressive PCR amplification combined with limited PCR extension time achieves library amplification and size selection at the same time (Figures 3, 4 and 5). Size selection is achieved because standard Taq DNA polymerase extension speed is on the order of 700–800 nucleotides per minute at 72°C. Instead of time-consuming gel-cutting for size selection, optimized progressive PCR can be performed to amplify and size-select libraries. We set the 72°C extension time to 30 seconds for 150–400 bp libraries. We used 18 cycles of PCR amplification cycles, then transferred plates immediately to 4°C (and never froze samples because extra PCR cycles may be required without adding any fresh enzyme or dNTPs). Next, we ran a small aliquot of the PCR product (5 μL) and verified the products on an agarose gel with EtBr. When a smearing range from 200–500 bp is nearly visible a minimal amplification condition has been achieved (Figure 3D). If there is no smear visible at all, the complete PCR reaction is returned to the thermal cycler and amplified for an additional 2–4 cycles, hence progressive PCR.

### Quality control check of final RRBS libraries

**Figure 6:**
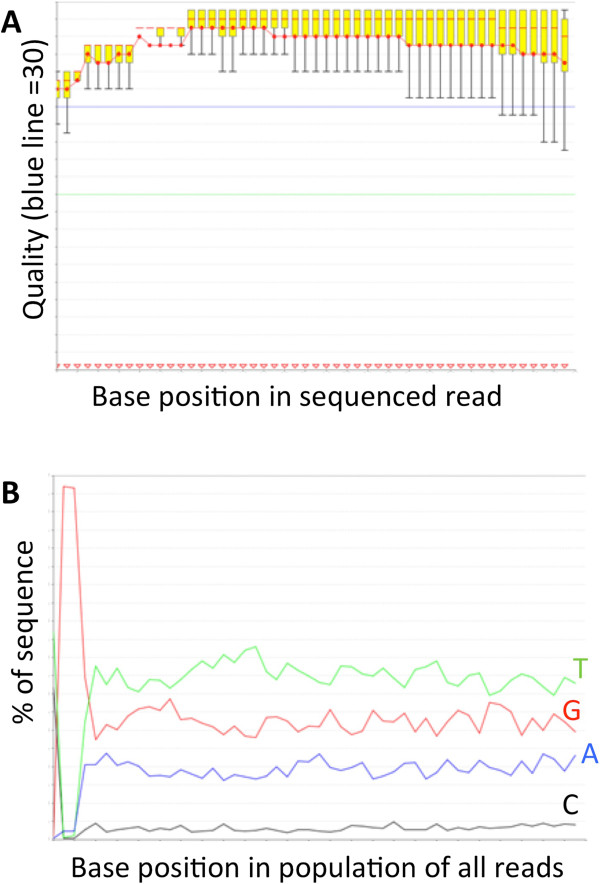
**Illumina HiSeq2000 QC of one representative sample. A)** Raw quality scores (FASTQ) of reads from one bisulfite treated library (G12; lane 6). **B)** Graph showing the representation of each base at each position in a 50 base read. Notice the very low level of Cytosine residues compared to the high content of Adenosine residues; non-bisulifte converted DNA shows approximately equal base composition at each site. One metric of C-T conversion rate is the ratio of C residues at bases 1–10 and 40–50. In the graph above, this ratio is about 1, as expected. In RRBS libraries, a large increase in Guanidine at positions 2 and 3 (~95% of reads have GG at these positions) should also be observed.

We used cloning and Sanger sequencing to verify the quality of bisulfite conversion as well as library quality (Figure 5). qPCR is used for absolute quantification of the final library and also serves as a check-point for the incomplete bisulfite conversion. As illustrated in Figure 5B, the dissociation curve from a bisulfite-converted RRBS libraries demonstrates a much lower melting temperature (5–8°C lower) as compared to a non-RRBS library (in this case, the qPCR standard PhiX library). This drop in the template melting temperature is caused by the excessive presence of T and A bases in the RRBS libraries. This is not meant as a quantification of conversion rate, but rather as a ‘sanity’ check - a large shift should be observed in the melting temperature at this step when bis-DNA versus non-bis libraries are compared. We performed real-time PCR using an ABI7000 SDS system (Applied Biosystems) with SYBR Green Master Mix according to the manufacturer’s instructions. Primers used were PE-qPCR_F and R (Table 1). Figure 6 demonstrates a raw Illumina QC output from one representative sample. Note the general quality of the library (Figure 6A; mean phred scores are above 30 at all positions), and the low cytosine content and approximately equal cytosines at positions 40–50 as compared to positions 1–10 (Figure 6B). Note also that >95% of reads have a large spike at base position 2 and 3 of a Guanidine dinucleotide. This is caused by the MspI digestion which is expected in any MspI RRBS library.

### Descriptive characteristics of methylation patterns across samples post-sequencing but pre- alignment

**Figure 7:**
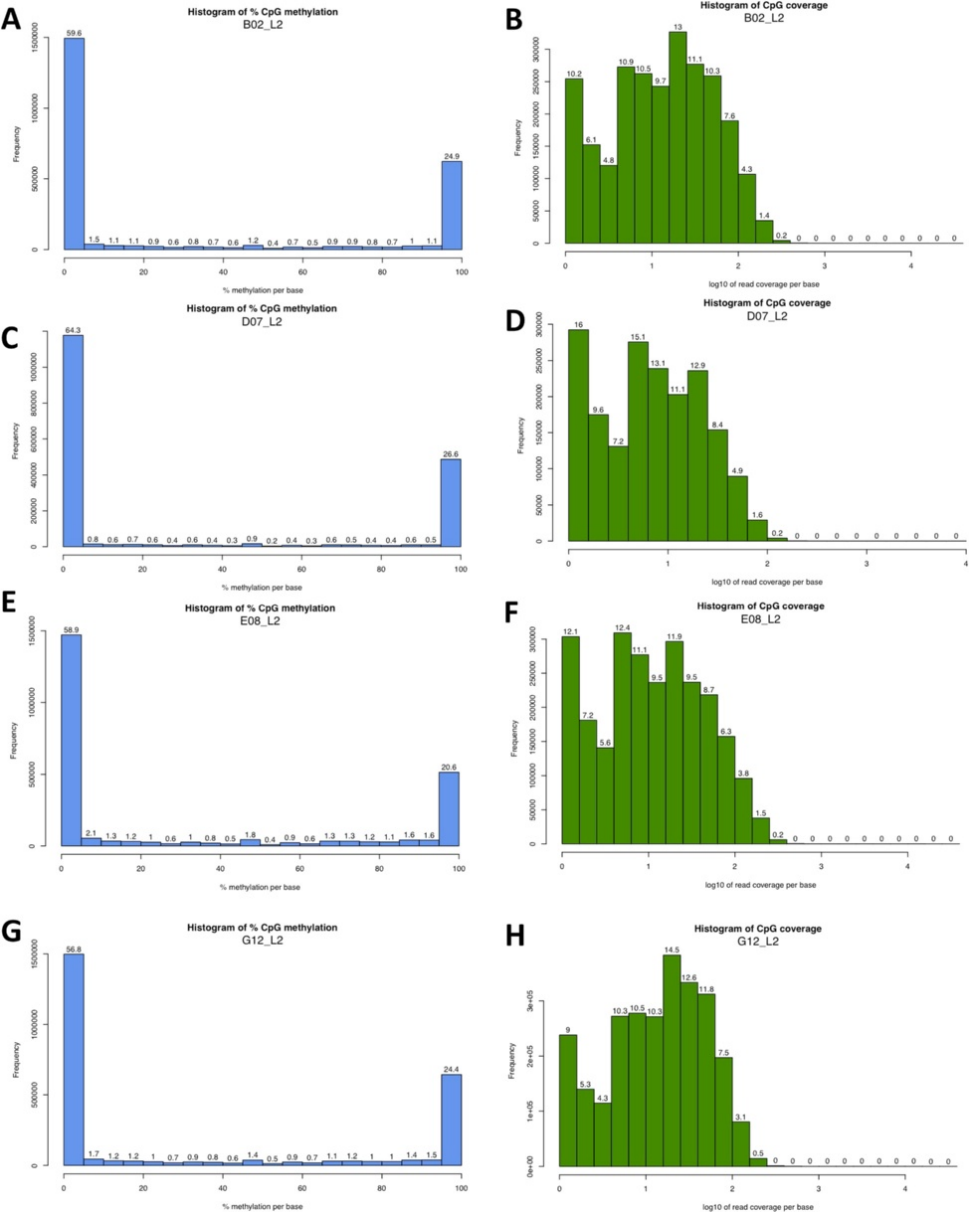
**Frequency of methylation per CpG and mean CpG coverage as QC metrics. A, C, E, G)** The frequency of methylation at any CpG region has very similar patterns across samples (A, C, E, G). In adult human brain tissue, >50% of all CpGs show 0% methylation and >20% of CpGs are fully methylated. A minority of CpGs lies within the extremes. **B, D, F, H)** Histograms denote average coverage per CpG in bins and where the X-axis is on the log_10_ scale (1 = 10X coverage). The purpose of performing these analyses is to assess if any sample deviates substantially from other samples. If so, these samples should be carefully re-investigated or re-processed. Here, the histogram pattern across samples B, D, F, and H are very similar.

Prior to beginning any experiment, a visual inspection of CpG distribution can be done. Even if an explicit hypothesis is being tested, the distribution of methylated to unmethylated CpGs is expected to be similar, and major deviations from this can be flagged in BisSeq experiments. For example, Figure 7A, C, E, G suggest that 55-65% of all CpGs have <5% methylation while 25-30% are methylated >95%, at least in DNA derived from human brain tissue. There is also a relatively consistent but low frequency of methylation between CpG residues methylated at 10%-89.99%, with a small but consistent increase when methylation data is binned in 5% blocks in the 45.0-49.99% methylated range. Methylation in this bin is usually 2- to 3-fold higher than neighboring bins (Figure 7A, C, E, G). Finally, coverage per CpG should be uniform across independent samples; compare Figure 7B, D, F, H (the log_10_ value of the coverage per CpG is consistent across independent experiments).

### Calculation of C-T conversion rate

The calculation of C-T conversion rate is a contentious issue in any bisulfite experiment, but is fundamental to accurate calling of methylation. Previous NGS studies using bisulfite converted DNA have reported conversion rates from 97-99% [12]. C-T conversion rates in the current study are 98.7-99.2% ± 0.1% across all 12 independent libraries. We calculate this conversion rate by adding the CHH and CHG variables from Bismark, where ‘H’ refers to any non-G base in the genome. It may be that methylation occurs at non-CpG sites in the human genome; convincing reports of this have begun to emerge [21,22] from mammalian genome studies. Normally an all ‘C’ DNA fragment could be included as a control in bisulfite sequencing studies, but this downplays the importance of the diversity of fragments in the genome which may effect conversion rates and has its own potential for errors being a homo-polymer. There are several checks that can be performed to assess C-T conversion: 1) an initial cloning experiment should be performed prior to NGS (Figure 3C) to determine how well C-T conversion has functioned, 2) an assessment of the C ratio in bases 1–10 and 40–50 in sequenced reads (the ratio should be approximately 1), and finally 3) an internal negative control can be used to assess C-T conversion (see Visualizing data in IGV and using the mitochondrial (MT) genome to assess C-T conversion).

### Visualizing data in IGV and using the mitochondrial (MT) genome to assess C-T conversion

**Figure 8:**
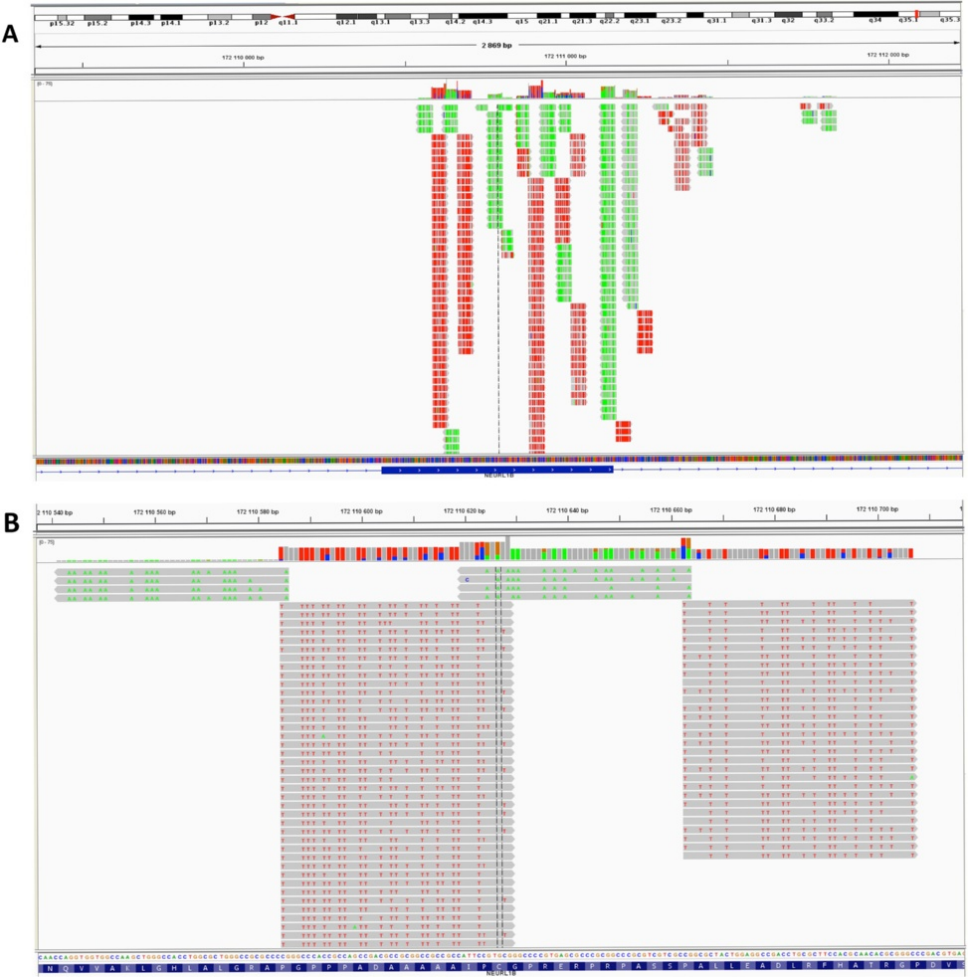
**Use of the Integrated Genome Viewer (IGV) showing an example of data at one locus in one subject. A**) IGV view of a 45 kb window on chromosome 5. Reads should map to distinct regions (due to MspI digestion), and there should be an equivalent balance of forward and reverse reads. Gray shading represents perfect alignment with the HG19 standard human reference genome. **B)** Close-up of the region from A) showing that forward reads have a high proportion of red ‘T’s and reverse reads have a high proportion of green ‘A’s; IGV shows colored bases in gray reads when an error is detected. In this case, the read is perfectly aligned to the standard reference genome, but, due to bisulfite conversion, most ‘C’s have converted to ‘T’s (forward strand), and this is outputted in the IGV window. Diversity at a locus is partially generated by different methylation levels at specific CpG sites and sequencing errors.

Visualization of aligned reads in the Integrated Genome Viewer (IGV) is a simple method to assess initial accuracy of alignment. Reads should begin at CGG or TGG (in both forward and reverse directions – see Figure 2), there should be equal balance between forward and reverse reads, and the number of non-methylated related errors should be what was set in the alignment program (usually <2 mismatches). Figure 8 shows an example of what this may look like for one region, but simply looking at different regions is inefficient, so we propose that the mitochondrial genome be used for initial visualization. Mammalian MT genomes are not methylated [23,24] and have extremely low CpG content [25], thereby providing an internal negative control for DNA methylation experiments. Methylation in the MT genome has also been recently confirmed to be absent from brain tissue [26] which is the tissue source used here. Specifically, alignment to the 16.6 Kb human MT genome allows for an assessment of C-T conversion rate (no C’s are expected to remain post-bisulfite conversion). Analyzing reads that align to the MT genome provides an isolated genomic locus with which to assess how well the alignment procedure has worked.

### Using imprinted regions and cloning to finalize alignment and library quality assessment

There are many known imprinting clusters in the human genome, though the tissue specificity and extent of methylation at each site are not precisely known [27,28]. To provide reference maps for expected methylation levels in human brain tissue and to demonstrate how these regions can be used as positive controls for a BisSeq experiment, we began by selecting known imprinted CpG islands previously documented in sperm [29]. Next, we assessed the same CpG islands in our sample using reads across all lanes and combining the data for each subject, though we included two CpG islands from genes that should not be imprinted, in other words, two regions where we did not expect to observe any methylation. Table 2 shows the results from this experiment and we note the relatively narrow ranges across subjects for each locus. To demonstrate this narrow range across subjects we show the mean and standard deviation for each locus (*IGF2R*:50.8% ± 4.7%, *GRB10*:26.3% ± 1.9%, *PEG3*:36.7% ± 6.3%, *MEST*:35.0% ± 1.5%, *RB1*:59.7% ± 2.2%, *KCNQ1*:37.4% ± 1.3%, *GAPDH*:1.3% ± 0.5%, *ACTB*:1.0% ± 0.5%). Table 3 shows the number of CpGs in the CpG island that were covered ≥ 1X and the mean coverage per CpG.

**Table 2:** Average methylation percentage across several known imprinted genes, with GAPDH and ACTB serving as comparison regions

**Gene**	***IGF2R***	***GRB10***	***PEG3***	***MEST***	***RB1***	***KCNQ1***	***GAPDH***	***ACTB***
**(CpG island location, hg19)**	**(chr6:160426265–160427502)**	**(chr7:50849753–50850871)**	**(chr19:57351284–57351995)**	**(chr7:130130740–130133111)**	**(chr13:48892636–48893857)**	**(chr11:2720411–2722087)**	**(chr12:6643262–6644607)**	**(chr7:5569063–5570594)**
**B02**	46.4%	24.8%	38.0%	34.4%	60.9%	39.2%	1.4%	0.8%
**D07**	50.0%	28.5%	44.7%	33.9%	61.2%	36.9%	0.7%	0.4%
**E08**	57.4%	27.2%	34.1%	37.2%	56.4%	36.4%	1.8%	1.7%
**G12**	49.4%	24.5%	30.1%	34.3%	60.1%	36.9%	1.3%	1.0%
**Sperm **[29]	69.8%	45.2%	42.0%	49.9%	57.7%	50.4%	-	-

**Table 3:** Number of CpG sites within a given CpG island observed and mean coverage per sequenced CpG

**Gene**	***IGF2R***	***GRB10***	***PEG3***	***MEST***	***RB1***	***KCNQ1***	***GAPDH***	***ACTB***
**(CpG island location, hg19)**	**(chr6:160426265–160427502)**	**(chr7:50849753–50850871)**	**(chr19:57351284–57351995)**	**(chr7:130130740–130133111)**	**(chr13:48892636–48893857)**	**(chr11:2720411–2722087)**	**(chr12:6643262–6644607)**	**(chr7:5569063–5570594)**
	**CpG/average coverage**	**CpG/average coverage**	**CpG/average coverage**	**CpG/average coverage**	**CpG/average coverage**	**CpG/average coverage**	**CpG/average coverage**	**CpG/average coverage**
**B02**	38/21.6X	89/13.0X	24/29.6X	99/18.25X	46/22.0X	138/18.6X	109/21.4X	195/14.2X
**D07**	20/19.3X	48/10.8X	24/14.7X	78/15.2X	38/16.3X	77/12.5X	97/14.6X	145/10.1X
**E08**	35/25.7X	99/16.8X	25/24.8X	94/25.1X	46/29.0X	125/20.5X	116/19.5X	206/16.6X
**G12**	39/23.0X	101/14.0X	30/21.3X	96/18.3X	46/29.1X	130/18.9X	121/19.5X	208/15.0X

**Figure 9:**
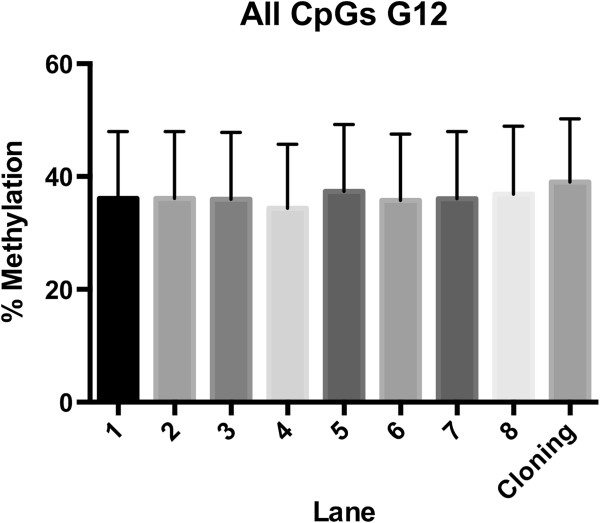
**Comparison of a single locus from one subject (G12) in reads from 8 different lanes and from an independent cloning experiment.** Average methylation per CpG in Illumina output data and cloned reads suggest the same methylation levels. This is one method to assess methylation calling in BisSeq computational pipelines.

Assessing imprinted regions for methylation status is an important check to ensure appropriate mapping (i.e., if percentages deviate significantly form an expected norm, data should be re-assessed), but a gold standard experiment to determine if methylation mapping and percentages are consistent with informatics analysis can also be done. To perform this experiment, we searched for high coverage regions with a mid level of methylation. We avoided regions with either 100% or 0% methylation because these likely would not show the variation across cell DNA that would be needed to test if the computational algorithm was able to accurately detect methylation patterns. To validate the accuracy of our RRBS results we cloned a 250 bp amplicon from the original library preparation of sample G12, which was the only subject sequenced across all lanes. We sequenced 40 individual clones, of those 35 were high quality and aligned to the reference sequence. This region gave us information for 15 individual CpG pairs. The average methylation level across all CpGs was not different between any of the 8 independent lanes of Illumina sequencing, or the cloning and Sanger sequencing (Kruskal-Wallis (9, 4.58) p = 0.8013; Figure 9).

### Significantly increasing the proportion of non-bisulfite converted DNA has a minimal impact on read count from bisulfite-converted libraries

Spike-in of non-bisulfite converted libraries might improve the quality of sequencing reads of bisulfite treated libraries. The reason for adding non-bisulfite converted DNA is that it can increase the performance of an MspI-based RRBS library by increasing the diversity of the first three bases. In theory, all MspI based RRBS library reads start with CGG or TGG (see Figure 2 for why this is) and this can cause a diversity issue on the Illumina HiSeq platform and result in incorrect base calls and lower QC scores. One way to address this issue is to diversify the first 3 bases in DNA fragments by the addition of non-bisulfite converted libraries which have a balanced A, C, G, T ratio.

To determine a reasonable concentration of non-bis DNA to incorporate we used identical libraries across 5 lanes with varying levels of non-bisulfite-converted PhiX library in 1%, 10%, 20%, 30%, or 50% increments. Table 4 shows the experimental design and Tables 5 and 6 show the output data from this experiment for all samples across all lanes. Additional file 1: Table S1 shows clustering information. We first asked if the quality call per base was different when different amounts of PhiX spike-in were used. The mean quality score for all bases from bisulfite converted libraries (N = 4) across each of the 5 lanes was (1% PhiX: 37.58 ± 0.03, 10% PhiX: 37.56 + 0.08, 20% PhiX: 37.44 ± 0.1, 30% PhiX: 37.18 ± 0.07, 50% PhiX: 36.42 ± 0.07), which corresponds to a linear, highly significant (p < 0.01 for all comparisons) decrease in quality as PhiX spike-in concentration increased; Importantly, all of these quality scores are excellent (mean phred score >35).

**Table 4:** Pooling and spike-in design for the analysis of the effects of non bisulfite-converted DNA pooled with bisulfite-converted DNA

	**Lane**	**1**	**2**	**3**	**4**	**5**	**6**	**7**	**8**
	**# of libraries**	4*	4*	4*	4*	4*	**1**	**6**	**12**
	**Non bisulfite-converted DNA proportion**	**1%**	**10%**	**20%**	**30%**	**50%**	**20%**	**20%**	**20%**
	1							B1	B1
	2	A2	A2	A2	A2	A2		A2	A2
	3								C3
	4								E4
	5								F5
Index #	6								A6
	7	D7	D7	D7	D7	D7		D7	D7
	8	E8	E8	E8	E8	E8		E8	E8
	9							H9	H9
	10								C10
	11								D11
	12	G12	G12	G12	G12	G12	G12	G12	G12

*The same 4 libraries were used in lanes 1–5, 7 and 8.

**Table 5:** Output data from sequencing experiment using non-duplicate reads

		**Raw-input**	**Aligned input**	**Unique aligned**	**% Unique aligned**	**1X COV (# of CpGs)**	**5X COV (# of CpGs)**	**10X COV (# of CpGs)**	**Mean COV**
Lane 1 **1%** non bis-converted DNA	B02	33 064 288	2 855 963	2 010 850	6.08	2 567 012	349 168	40 326	2.63
	D07	22 138 393	1 819 725	1 255 728	5.67	2 062 303	121 179	6 308	1.98
	E08	27 604 275	3 277 855	2 323 092	8.41	2 566 936	527 206	109 576	3.27
	G12	34 606 411	3 331 718	2 318 284	6.69	2 714 010	422 645	47 241	2.81
Lane 2 **10%** non bis-converted DNA	B02	35 305 177	2 951 835	2 078 732	5.89	2 567 438	380 661	48 125	2.73
	D07	13 678 215	1 406 910	999 858	7.31	1 870 722	63 063	2 673	1.77
	E08	29 182 244	3 343 563	2 371 936	8.13	2 564 006	549 654	119 076	3.35
	G12	35 675 527	3 377 167	2 352 532	6.59	2 706 283	443 966	50 919	2.87
Lane 3 **20%** non bis-converted DNA	B02	29 189 642	2 680 090	1 903 402	6.52	2 506 260	315 769	33 092	2.55
	D07	11 434 385	1 281 232	1 281 232	11.2	1 798 599	47 005	1 960	1.69
	E08	29 178 605	3 315 119	2 354 427	8.07	2 555 851	543 668	117 267	3.33
	G12	32 077 566	3 190 847	2 234 841	6.97	2 672 851	396 964	41 721	2.75
Lane 4 **30%** non bis-converted DNA	B02	26 075 222	2 601 566	1 853 844	7.11	2 471 169	302 050	30 356	2.52
	D07	15 915 092	1 563 145	1 101 459	6.92	1 923 817	86 567	3 720	1.87
	E08	22 095 982	2 896 518	2 075 605	9.39	2 456 950	446 280	80 977	3.04
	G12	27 526 678	3 034 738	2 139 571	7.77	2 629 556	363 919	35 701	2.68
Lane 5 **50%** non bis-converted DNA	B02	20 542 906	2 606 323	1 841 922	8.97	2 368 596	321 222	32 684	2.62
	D07	9 506 379	1 299 321	925 451	9.74	1 716 465	57 531	2 107	1.78
	E08	18 652 337	2 828 208	2 007 876	10.76	2 359 943	440 954	81 427	3.07
	G12	23 046 688	3 069 911	2 146 208	9.31	2 559 434	391 118	36 982	2.77
Lanes 6 **20%** non bis-converted DNA	B02					-			
	D07					-			
	E08					-			
	G12	147 537 115	6 665 204	4 381 541	2.97	2 988 624	1 086 692	383 384	4.93
Lane 7 **20%** non bis-converted DNA	B02	20 450 646	2 241 773	1 614 966	7.9	2 395 119	213 017	15 790	2.26
	D07	8 334 712	1 087 639	791 533	9.5	1 671 805	27 718	1 338	1.57
	E08	19 548 711	2 663 849	1 915 409	9.8	2 415 658	385 375	62 823	2.85
	G12	21 853 857	2 654 189	1 886 840	8.63	2 562 616	267 113	20 703	2.42
Lane 8 **20%** non bis-converted DNA	B02	10 420 846	1 644 906	1 213 820	11.65	2 156 458	95 891	4 665	1.89
	D07	4 061 039	774 145	578 180	14.24	1 394 239	8 729	914	1.39
	E08	6 950 628	1 494 036	1 105 554	15.91	1 994 770	113 007	9 195	1.98
	G12	11 162 134	1 928 010	1 405 997	12.6	2 334 809	120 111	6 110	1.98

**Table 6:** Output data when including duplicate reads

**Lane**	**Sample**	**Raw-input**	**Alignment-input**	**Unique aligned**	**% Unique aligned**	**1X COV (# of CpGs)**	**5X COV (# of CpGs)**	**10X COV (# of CpGs)**	**Mean COV**
**1**	B02	33 064 288	29 063 371	20 255 109	61.26	2 567 012	1 918 910	1 530 535	29.79
	D07	22 138 393	17 645 024	11 939 603	53.93	2 062 303	1 389 067	1 058 733	20.73
	E08	27 604 275	24 916 571	17 486 794	63.35	2 566 936	1 802 043	1 381 774	27.65
	G12	34 606 411	30 925 499	20 902 905	60.4	2 714 010	2 109 753	1 713 530	28.79
**2**	B02	35 305 177	29 236 310	20 364 338	57.68	2 567 438	1 917 970	1 532 194	29.99
	D07	13 678 215	10 201 505	6 940 443	50.74	1 870 722	1 147 585	781 304	13.52
	E08	29 182 244	24 797 995	17 379 826	59.56	2 564 006	1 795 028	1 374 048	27.53
	G12	35 675 527	30 029 857	20 267 725	56.81	2 706 283	2 091 749	1 691 941	27.99
**3**	B02	29 189 642	23 540 460	16 370 890	56.08	2 506 260	1 806 333	1 393 509	24.61
	D07	11 434 385	8 327 312	5 657 862	49.48	1 798 599	1 043 794	667 799	11.41
	E08	29 178 605	24 189 204	16 950 994	58.09	2 555 851	1 770 930	1 349 999	26.88
	G12	32077566	26 281 812	17 702 331	55.18	2 672 851	2 022 443	1 596 346	24.60
**4**	B02	26 075 222	21 021 219	14 594 149	55.97	2 471 169	1 740 262	1 313 326	22.17
	D07	15 915 092	11 619 325	7 851 241	49.33	1 923 817	1 191 959	832 127	14.57
	E08	22 095 982	18 227 193	12 761 292	57.75	2 456 950	1 611 115	1 170 745	20.94
	G12	27 526 678	22 602 833	15 277 998	55.5	2 629 556	1 938 216	1 484 737	21.55
**5**	B02	20 542 906	15 122 524	10 444 122	50.84	2 368 596	1 533 603	1 268 102	16.48
	D07	9 506 379	6 102 317	4 110 762	43.24	1 716 465	876 141	498 166	8.65
	E08	18 652 337	14 027 421	9 789 610	52.48	2 359 943	1 437 779	995 396	16.70
	G12	23 046 688	17 307 795	11 663 692	50.6	2 559 434	1 764 888	1 268 102	16.86
**6**	B02	-	-	-	-	-	-	-	-
	D07	-	-	-	-	-	-	-	-
	E08	-	-	-	-	-	-	-	-
	G12	147 537 115	99 359 722	66 638 301	45.17	2 988 624	2 499 292	2 279 640	82.78
**7**	B02	20 450 646	15 984 580	11 124 200	54.4	2 395 119	1 596 904	1 139 946	17.50
	D07	8 334 712	5 747 869	3 899 752	46.79	1 671 805	859 372	476 623	8.47
	E08	19 548 711	15 949 864	11 173 920	57.16	2 415 658	1 540 771	1 093 107	18.75
	G12	21 853 857	17 549 017	11 807 885	51.13	2 562 616	1 799 635	1 299 192	17.11
**8**	B02	10 420 846	8 057 402	5 615 587	53.89	2 156 458	1 171 935	690 974	9.81
	D07	4 061 039	2 785 294	1 891 770	46.58	1 394 239	493 022	177 403	4.913
	E08	6 950 628	5 557 934	3 889 196	55.95	1 994 770	906 953	490 601	7.87
	G12	11 162 134	8 765 612	5 907 141	52.92	2 334 809	1 324 775	759 389	9.39

We next determined what the total number of achievable reads was for an experiment; here we used one full lane to sequence one bisulfite-converted library (Table 5; lane 6). We found 4.38 M unique alignable reads in this analysis, with a mean coverage of 4.93X per CpG (coverage definition to follow), using only non-duplicate reads. Given that there was ~ 150 M reads per lane, sequencing one subject per lane is very inefficient; however, there is a logarithmic relationship between number of reads and number of unique alignable reads in an RRBS experiment. That is, there are diminishing returns as one continues to sequence the same sample – largely due to the high number of duplicates in each library.

We estimate that approximately >2 M unique reads is the range where ‘returns’ (unique reads) significantly diminishes. This is best demonstrated by the samples that were fixed across multiple lanes, but with decreasing concentration as the proportion of non-bis DNA increased. We take one sample from Table 5 to demonstrate this: sample G12 yielded 2.31 M, 2.35 M, 2.23 M, 2.14 M, and 2.14 M reads in lanes 1 to 5, respectively, despite the fact that the proportion of this library represented just 24.75%, 22.5%, 20%, 17.5%, and 12.5% of each lane, respectively. One would normally expect a decrease in unique, alignable reads between the first and last points to be 50%, yet in fact the decrease is just 7.5%. This relationship was confirmed in three other independent libraries (Table 5), suggesting that this is not an artifact of a single library or of liquid handling. Our data further suggest that one major reason for this is the increase in cluster density as non-bis increases (Additional file 1: Table S1). Cluster density is directly proportional to the total number reads, meaning that the more clusters there are the more reads there will be.

Given that the total number of usable reads does not change substantially (even when the proportion of a lane comprising bisulfite-converted libraries is decreased by 50%) with increasing proportion of non-bisulfite converted DNA, we would suggest that bisulfite converted libraries can be run with non-bisulfite converted libraries at minimal cost to sequencing output. This has significant implications for cost savings and strongly encourages genome centers or individual labs to save sequencing costs by pooling bis DNA and standard DNA libraries in the same lane. We currently use this approach (i.e., using non bisulfite-converted DNA not from PhiX but from the complete human genome) for on-going projects.

### Calculation of coverage and use of duplicates in coverage statistics

**Figure 10:**
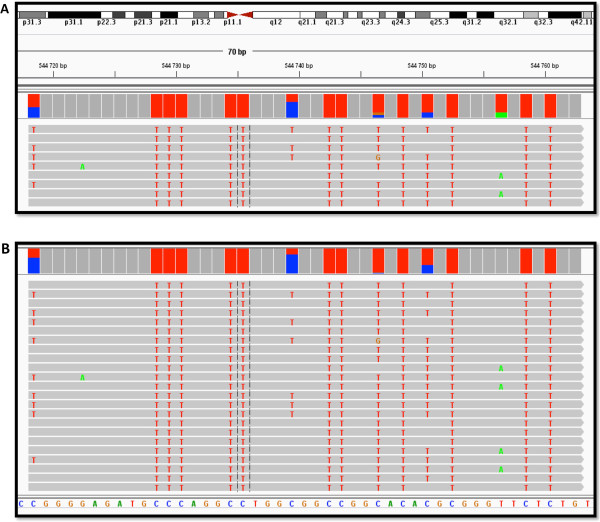
**A method to validate the inclusion of duplicate reads for coverage statistics.** Individual reads where no duplicates are present **(A)** and where duplicates are included **(B)**. Duplicates can be used in coverage statistics if the proportion of methylation in the duplicate read pool is significantly correlated to the non-duplicate read pool. In the locus shown here, there are four sites that are differentially methylated and the proportion of methylation in the non-duplicate and duplicate pools are similar.

A genomic library with reduced representation from the genome (due to targeted enzymatic digestion) and reduced read complexity (due to bisulfite-mediated C-T conversion) leads to a situation where many identical reads may be observed but which come from independent DNA sources (i.e., not from PCR amplification). To highlight this, we demonstrate coverage over one locus in Figure 10. First, note that unique read coverage comes from 1 of 4 possibilities; 1) from read ‘pile-up’ over a given region where MspI digestion sites are close, so that the same CpG is covered by different fragments, 2) by reads with identical MspI digestion sites but which have acquired errors, so map to the locus but are derived from a different read cluster, 3) by reads with identical MspI digestion sites, but with different methylation patterns, so map to the same locus, and 4) sequencing reads from the reverse strand. Reads with a small number of errors (usually set at 2 in Bismark) are highly useful in this context. In standard sequencing libraries, coverage is determined by pile-up, where reads have different start and end regions. Here, reads at a given locus mostly have identical start and end points, due to targeted enzymatic digestion and Illumina-determined read size (here, 50 bases). This suggests that regions of very high or low methylation will have lower coverage in these libraries (extremely high or low methylation patterns will lead to identical reads).

How should coverage of a bisulfite-converted library be determined, given that enzymatic digestion gives identical fragment sizes when representation of the genome is reduced? A conservative approach is to use unique reads only and to calculate coverage over CpG sites only. In our data this approach gives a mean coverage of approximately 2-4X (Table 5). Because this is largely seen as inadequate to call methylation differences, different groups have included duplicates in their coverage calls, but duplicates are not normally used in coverage statistics from massively parallel sequencing experiments. The importance of utilizing duplicates is that they function as proxies to the true number of fragment reads, implying that simply filtering out duplicates removes valid data; however, as of yet there is no metric to determine when or how duplicates should be used. Table 6 shows how output data from the experiment shown in Table 5 changes when duplicates are included - coverage increases by ~10-fold (from 2-4X to 20-30X). The relationship between methylation frequency calls and coverage may also be a limitation of RRBS more generally; that is, that low sampling of unique reads is inevitable.

We reasoned that if duplicates are valid to use they should be representative of the pool of unique reads. To determine if duplicate reads are representative of the unique aligned reads, we wanted to assess a sample of genomic loci where there are many unique reads (for power) and where a given read has a high number of CpGs. ‘Representativeness’ can be assessed in different ways, but the issue we most wanted to test was whether PCR bias (from library amplification) led to poor representation. PCR bias (where different fragments are amplified preferentially over others) occurs for two independent reasons in BisSeq libraries: 1) fragments derived from the same locus can have different methylation patterns; increased methylation leads to the preservation of cytosine residues which have different melting temperatures, different interactions with polymerase, and different annealing times than fragments with ‘T’ residues; 2) 50 bp SE sequencing does not alter the fact that libraries were selected at 150–400 bp in the progressive PCR step. Shorter fragments may be amplified preferentially than longer ones because annealing and extension times are shorter. Because the polymerase extension time is fixed in the library amplification step, longer fragments may not amplify in the initial PCR stages, but all short fragments will. PCR amplification can lead to a BisSeq library that includes duplicates in the mapping statistics being unrepresentative of the initial BisSeq library.

To assess this idea, we calculated a Pearson correlation between methylation sites in all unique aligned reads compared to all reads at the same genomic locus. Figure 10 shows an example region to demonstrate this concept. Figure 10 and Table 7 show four sites with differential methylation at one locus, (10 A no duplicates; 10 B with duplicates). Using just four CpG sites at this region selected for high CpG coverage in uniquely aligned reads, we found an r^2^ value of 0.95 between reads from the uniquely aligned reads and all reads, which has a p-value of 0.03 with a CpG N of four. While all the reads present in the uniquely aligned group are present in the all read group, this Pearson calculation helps to guard against potential PCR bias (i.e., over-amplification of certain library fragments over others). If one DNA fragment happens to amplify at a significantly higher rate than any other, the Pearson correlation coefficient would drop and duplicates would then be less representative of non-duplicate, uniquely aligned reads.

Since pile-up of non-duplicate reads is low at any given locus and this QC metric requires differential methylation at CpG sites in the same read, we recommend assessing all loci with several unique reads (>10) and differential methylation (>four differentially methylated sites). Significant r^2^ values at these regions would suggest that the library constructed is of good quality, and that duplicates can be used for assessing coverage statistics for the library.

**Table 7:** Correlation of the frequency of methylation at a single locus between reads with duplicates and reads without duplicates

**CpG**	**Without duplicates (9 reads)**	**With duplicates (23 reads)**
1	0.44	0.61
2	0.67	0.74
3	0.11	0.043
4	0.22	0.22

These data sets correspond to the graphic in Figure 10. This Table shows the frequency of methylation at each CpG in the read grouping where duplicates are removed and where duplicates remain. The r^2^ value associated with these data is 0.95 (N = 4, p = 0.03).

## Discussion

We have proposed a set of improvements, from library construction to bioinformatic analysis, for any experiment using bisulfite converted DNA for the purpose of methylation mapping, and addressed two issues in bisulfite sequencing experiments, namely the effect of including non-bis DNA in BisSeq experiments, and the extent to which duplicate reads can be used to calculate coverage statistics. Our detailed approach and suggestions for sample pooling and coverage calculations should be useful in any experiment that uses massively parallel sequencing to understand DNA methylation states.

We have several quality control checkpoints at each stage of the process, including library construction, pre-sequencing, post-sequencing, and post-alignment. At the library construction stage, we propose a multiplexing approach that uses a post-library indexing step, which allows the user to select an indexing strategy after library synthesis. Because this technique also involves smaller adaptors, it allows for simple sample clean-up using inexpensive, efficient spin-columns. The progressive PCR step for library amplification allows fragment size selection by altering PCR extension time while allowing the user to achieve the lowest possible cycle number per library. Together, these improvements simplify and speed-up the library synthesis process. At the pre-sequencing stage, we recommend cloning a small number of fragments to ensure fragment diversity (there should be no identical clones) and to assess C-T conversion. A qPCR experiment can also be done using primers directed at adaptor ends and compared to melting curves for standard libraries. While this is a gross measure – 5% differences bisulfite conversion are unlikely to be detected for example, it is a simple check to reassure, particularly since qPCR experiments are often required for accurate quantification of library concentration prior to sample pooling (multiplexing).

There are several assessments that can be done immediately after massively parallel sequencing but before alignment. Assessing base composition in sequenced reads will give a further reassurance about bisulfite conversion, for example. If there is a higher proportion of cytosine bases at position 40–50 in sequenced read compared to bases 1–10, this may be evidence of incomplete conversion or sequencing error; one expects equal representation of all bases across all positions, with significantly lower cytosine residues compared to the other bases. In an RRBS experiment, the strong majority of reads should have CGG or TGG at base position 2, 3, and 4. If this is not observed, it suggests a problem with the enzymatic digestion. Post-alignment, we generated a novel computational tool to make the Bismark output readable in the IGV. We suggest assessing all reads in the <17Kb MT genome to determine library quality: are reads the size that is expected? Is there equal representation from forward and reverse reads? Are all C residues converted to T? Beyond visual assessments, we also generated data at several imprinted regions that can be used by any other group using brain DNA to determine library quality. Imprinted regions are expected to have a specific level of methylation and if these regions show a large deviation from what is expected, it may suggest alignment or other problems. We also used molecular cloning to determine if our methylation calling parameters were accurate. Having two different technologies (NGS and cloning) show strongly similar methylation patterns in a given region suggests that computational calling parameters are accurate.

A further goal of this project was to understand the role of pooling non-bis DNA with bis-DNA. We found that increasing the proportion of non-bis DNA libraries with bis-DNA libraries led to a minimal decrease in uniquely aligned reads and significantly increased cluster density, suggesting that pooling unrelated samples that have not undergone bisulfite conversion is not only viable but recommended to decrease sequencing costs. The increased proportion of reads that were sequenced when bis DNA was decreased in a lane was due both to sequencing less duplicates from a library and to increasing clustering density presumably created by the addition of more diverse DNA fragments. Because duplicates are desirable in a well-performed BisSeq experiment, we recommend a 30% spike-in of non-Bis DNA. A 30% spike-in will give almost identical CpG coverage as at 1% spike-in, and allow for the sequencing of unrelated non-bis DNA samples.

We also addressed the validity of using duplicates in calculating coverage for BisSeq experiments because duplicate reads are traditionally discarded in massively parallel sequencing experiments. We suggested that PCR bias can be assessed by using a correlation assessment where a single locus has many CpGs and many unique reads. By comparing CpG methylation frequency in the non-duplicate read pool to the all-read pool, one expects to observe a strong correlation between pools. A low correlation might be evidence of over-amplification in the library preparation stage which will be biased towards shorter DNA fragments and fragments with less cytosine residues.

Where does the future lie for methylation mapping? A move from RRBS to whole genome bisulfite sequencing (WGBS) has already begun, though these whole genome experiments are much more expensive and still have significant alignment issues. The fractionation of DNA instead of the enzymatic digestion improves things considerably because most fragments will be generated from different genomic locations instead of from CCGG locations. Still, it is not clear that WGBS is worth the cost given that sites rich in CpGs continue to be assessed, and these are covered to a wide extent in RRBS, though recent evidence from mouse suggests that the most functionally relevant DNA methylation sites might be located in CpG poor distal regulatory regions with low methylation levels. In this case, WGBS will be preferable because RRBS will miss many of these sites because of the absence of CCGG at the CpG poor distal regulatory regions [30]. It is clear that bisulfite sequencing is not an ideal methodology but it is currently the best available option to give reproducible data. Bisulfite treatment of DNA causes DNA damage and recent reports suggest that hydroxymethylation may also be an important epigenetic mark [31] and this may confound BisSeq experiments because hydroxymethylation and methylation are indistinguishable after bisulfite treatment and PCR. Single molecule sequencers [32], whereby unamplified and untreated ‘native’ DNA is sequenced are not yet ready for prime-time; however, it is foreseeable that all methylation (or indeed epigenetic mark-mapping) is done on single molecule sequencers in the not-to-distant future [33].

## Conclusion

These data suggest improved ways to process and analyze bisulfite sequencing data. We provide new methods for library construction and indexing, as well as several new options for QC assessment as well as recommendations for generating high quality data.

## Availability of supporting data

All supporting data is available within this manuscript and at the Ernst lab website at:http://www.mcgill.ca/psychiatricgenetics.

## Abbreviations

Bp: Base pair; EtBr: Ethidium Bromide; MT: Mitochondrial; NGS: Next Generation Sequencing; RRBS: Reduced Representative Bisulfite Sequencing; WGBS: Whole Genome Bisulfite Sequencing.

## Competing interests

The authors declare that they have no competing interests.

## Authors’ contributions

CE designed experiments and conceived of the study. GC carried out experiments. CE and GC wrote the manuscript. AD, RJ and CE performed bioinformatic analyses. AS, CN, KV, PEL, and VO assisted with experiments. DM and GT supplied reagents. All authors read and approved the final manuscript.

## Supplementary Material

Additional file 1 Clustering informations, as well as adaptor and indexing primers fusion products, of BisSeq libraries Click here for file

## Ackowledgements

This work was funded by a research grant RGPIN 435512–2013 from the Natural Science and Engineering Research Council of Canada (CE) and PHS NIDA grant DA033684 (DM and GT). CE is supported by the Canada Research Chairs Program.
