# Supplementary material for: BisQC: an operational pipeline for multiplexed bisulfite sequencing
Source: BMC Genomics. 2014 Apr 16;15:290. doi: 10.1186/1471-2164-15-290 (PMC4234473; doi:10.1186/1471-2164-15-290)
Supplement: Additional file 1 — Clustering informations, as well as adaptor and indexing primers fusion products, of BisSeq libraries [file 1471-2164-15-290-S1.docx]

**Chen et al.**

**BisQC: An operational pipeline for multiplexed bisulfite sequencing**

**Supplemental text:**

Using the Illumina two-step indexing approach, where the index is not a part of the adaptors themselves (which is the one-step approach, and the current Illumina design), can create novel products that are not filtered out by trimming adaptor sequences. This is because the indexing primer can ligate to the paired-end adaptor and create a novel product. In the current dataset, we most frequently observed the following fragments that are a result of this process:

TGGTGATCGGAAGAGCGGTTCAGCAGGAATGCCG

TGGATCGGAAGAGCGGTTCAGCAGGAATGCCG

These fragements can be detected using the TrimGalore algorithm, which can be downloaded here: (http://www.bioinformatics.babraham.ac.uk/projects/trim_galore/). We found these sequences in libraries at the following levels: 0.8% in E08, 1.8% in G12, 2.8% in B02, and 11.4% in D07, expressed as a percentage of total reads. In general, if libraries are of low concentration at the indexing step, a higher percentage of these fusion fragments are detected. These fragments also may have been incorporated into recent genome assemblies, and while they might be representative of the true genome it is possible that they are a sequencing artifact. The following warrant further investigation, for example:

Position 9 to 38

http://www.ncbi.nlm.nih.gov/nucleotide/440586614?report=genbank&log$=nuclalign&blast_rank=2&RID=HYMR6V7E014

and

Position 2828 to 2857

http://www.ncbi.nlm.nih.gov/nucleotide/549805986?report=genbank&log$=nuclalign&blast_rank=1&RID=HYMR6V7E014

**Supplemental Table S1.** **Overall raw statistics for the sample G12 from the Illumina HiSeq2000 sequencing run.**

| Output | Lane 1 | Lane 2 | Lane 3 | Lane 4 | Lane 5 | Lane 6 | Lane 7 | Lane 8 |
| --- | --- | --- | --- | --- | --- | --- | --- | --- |
| % phiX (target) | 1% | 10% | 20% | 30% | 50% | 20% | 20% | 20% |
| Multiplexing level | 4 | 4 | 4 | 4 | 4 | 1 | 6 | 12 |
| Raw cluster density (K/mm2) | 550 | 550 | 540 | 605 | 670 | 660 | 585 | 550 |
| PF cluster density (K/mm2) | 475 | 485 | 500 | 550 | 555 | 525 | 505 | 510 |
| Total # of Reads (M) | 34.6 | 35.7 | 32.1 | 27.5 | 23.0 | 147.5 | 21.9 | 11.2 |
| Avg Q score | 38 | 38 | 37 | 37 | 36 | 37 | 37 | 37 |
| % Duplicates | 94.1 | 94.1 | 93.6 | 92.9 | 91.2 | 97.2 | 91.9 | 87.7 |
| % of reads beginning with CGG | 40.0 | 37.6 | 37.2 | 36.7 | 32.2 | 30.4 | 36.4 | 35.4 |
| % of reads beginning with TGG | 55.9 | 52.6 | 50.6 | 51.2 | 48.2 | 41.9 | 49.7 | 48.8 |
| % of reads beginning with non-YGG | 4.1 | 9.7 | 12.2 | 12.0 | 19.6 | 27.7 | 13.9 | 15.8 |
| Non-duplicates (M) | 3.3 | 3.4 | 3.2 | 3.0 | 3.1 | 6.7 | 2.7 | 1.9 |
| Uniquely mapped (M)  (post-processing) | 2.3 | 2.4 | 2.3 | 2.1 | 2.2 | 4.4 | 1.9 | 1.1 |
